# Linking factors to incisional hernia following pancreatic surgery: a 14-year retrospective analysis

**DOI:** 10.1007/s10029-024-03067-z

**Published:** 2024-05-12

**Authors:** Nadav Nevo, Arielle Jacover, Eran Nizri, Diego Cuccurullo, Corrado Rispoli, Ron Pery, Yoav Elizur, Nir Horesh, Rony Eshkenazy, Ido Nachmany, Niv Pencovich

**Affiliations:** 1 Department of General Surgery and Transplantation, Sheba Medical Center, Tel-Aviv University, Tel-Aviv, Israel; 2 Department of Laparoscopic and Robotic General Surgery, Azienda Ospedaliera Dei Colli “Monaldi Hospital”, Naples, Italy; 3 General Surgery Division, Tel-Aviv Medical Center, Tel-Aviv University, Tel-Aviv, Israel; 4 Department of Internal Medicine B, Sheba Medical Center, Tel Hashomer, Tel-Aviv University, Tel-Aviv, Israel

**Keywords:** Hernia, Pancreatic surgery, Risk factors, Incisional hernia, Biliary stent, SSI, Pancreatic fistula

## Abstract

**Background:**

Incisional hernias (IH) are a significant postoperative complication with profound implications for patient morbidity and healthcare costs. The relationship between IH and perioperative factors in pancreatic surgery, with particular attention to preoperative biliary stents and pancreatic fistulas requires further exploration.

**Methods:**

This retrospective observational study examined adult patients who underwent open pancreatic surgeries via midline incision at a high-volume tertiary hepatopancreatobiliary center from January 2008 to December 2021. The study focused on IH incidence and associated risk factors, with particular attention to preoperative biliary stents and pancreatic fistulas.

**Results:**

In a cohort of 620 individuals undergoing pancreatic surgery, 351 had open surgery with at least one-year follow-up. Within a median follow-up of 794 days (IQR 1694–537), the overall incidence of IH was 17.38%. The highest frequency of IH was observed among patients who had a Pancreaticoduodenectomy (PD). Significant predictors for the development of IH within the entire study population in a multivariable analysis included perioperative biliary stenting (OR 2.05; 95% CI 1.06–3.96; p = 0.03), increased age at diagnosis (OR 2.05; 95% CI 1.06–3.96; p = 0.01), and BMI (OR 1.08; 95% CI 1.01–1.15; p = 0.01). In the subset of patients who underwent Pancreaticoduodenectomy (PD), although the presence of biliary stents was associated with a heightened occurrence of SSIs, it did not demonstrate a direct correlation with an increased incidence of incisional hernias (IH). The development of pancreatic fistulas did not show a significant correlation with IH in either the Distal Pancreatectomy with Splenectomy (DPS) or the PD patient groups.

**Conclusions:**

The study underscores a notable association between biliary stent placement and increased IH risk after PD, mediated by elevated SSI incidence. Pancreatic fistulas were not directly correlated with IH in the studied cohorts. Further research is necessary to validate these findings and guide clinical practice.

## Introduction

Incisional hernias (IH) are a significant concern in postoperative care, with a profound impact on patient morbidity, escalating healthcare costs, and the overall quality of life [1]. The etiology of IH is multifactorial, with patient age, comorbidities such as diabetes, immunosuppression, smoking, obesity and more playing a pivotal role. Factors such as type of incision, surgical technique during closure of the fascia, postoperative complications such as wound infection, and more, were also shown to be associated with the development of IH [2]. The nature of the procedure was shown to be crucial in determining the likelihood of future development of IH, and certain operations like permanent colostomy or abdominal aortic aneurysm repair particularly associated with IH occurrence [3, 4] In some cases, preoperative planning and preventive measures, such as weight loss or the prophylactic placement of a mesh, may mitigate this risk.

Despite the breadth of knowledge regarding IH, the specific challenges posed by pancreatic surgery have been relatively neglected [5, 6]. Partial pancreatic resection, which can be broadly divided to pancreaticoduodenectomy (PD) and left pancreactectomy (LP), is a complex field with various intricacies and perioperative and intraoperative considerations that may affect the likelihood of long-term development of IH. The effect of specific factor related to pancreatic surgery such as jaundice, preoperative stenting of the common bile duct (CBD), neoadjuvant therapy for pancreatic ductal adenocarcinoma (PDAC), as well as specific postoperative complications such as pancreatic fistula on development of IH is yet to be evaluated.

This study aims to bridge this knowledge gap by exploring the array of factors unique to pancreatic surgery that may influence IH incidence. Through this investigation, we seek to identify patients at higher risk for IH after pancreatic surgery and propose tailored strategies to improve their postoperative outcomes.

## Methods

### Patients

This retrospective study included all adult (over 18 years old) patients who underwent pancreas surgery at a specialized Hepato-Pancreato-Biliary (HPB) referral center during the period from January, 2008, to December 2021. Excluded were patients who underwent minimally invasive surgeries (mostly for none cancerous, small cystic lesions), those who were lost to follow-up, and those who did not have a minimum of 12 months of postoperative follow-up data available. All open pancreas surgeries in this study were performed via midline incision from the xiphoid process, circumventing the umbilicus, down to approximately 2 cm below the umbilicus. Patients with no prior CBD stenting were treated empirically with first generation cephalosporin antibiotics perioperatively for 24 h. Patients that underwent preoperative CBD stent placement were treated with third generation cephalosporins (Rocephin) and Metronidazol, or according to previously docymented bacterial growth, until negative results of intraoperative bile cultures. If specific bacteria were identified via intraoperative bile cultures, an antibiotic corresponding to the specific growth was continued for 5 days after surgery. We meticulously retrieved data from the electronic medical records of both the surgery and oncology departments. Data retrieval was conducted with the assistance of MDClone© software, a data extraction and synthesis tool intricately connected to the medical records of patients treated within our institution (http://www.mdclone.com). The comprehensive dataset encompassed various patient-related information, including demographics, medical history, comorbidities, lesion characteristics, perioperative laboratory indices, preoperative treatments, operative details, and postoperative outcomes, including the occurrence of complications. Special attention was directed towards the incidence of surgical site infections (SSIs), the utilization of preoperative biliary stents, the presence of pancreatic fistulas, and the use of neoadjuvant treatments. Postoperative complications were classified according to the Clavien-Dindo system and major complications defined as Clavien-Dindo > 3a [7].To ensure data accuracy and reliability, the information collected through MDClone underwent rigorous manual assessment and validation for all patients. The primary outcome measure was the development of an incisional hernia after at least 1 year following surgery. Incisional hernias were identified either through clinical examinations during routine follow-up visits or by radiological imaging, routinely performed to most of these patients as part of the oncological follow-up.

The study was conducted in accordance with the ethical guidelines and was approved by the Institutional Review Board at XXXXXX, with approval number SMC-9498-22. Informed consent requirement was waived by the ethics committee for this retrospective study.

### Statistical analysis

Continuous variables are presented as mean and standard deviation (SD) and categorical variables as number and percentage. Differences between groups were compared using the Mann–Whitney U test for continuous variables and Fisher’s exact tests for categorical variables. Patients with missing data values were excluded from calculation of means/percentages and statistical comparison for the respective variable. Two-sided p-values of less than 0.05 were considered statistically significant. All statistical calculations were performed using SPSS 29.

Predictors of incisional hernia within the pancreatic procedures cohort were evaluated using a multivariate logistic regression model which was conducted using the “Enter” method and included only variables found significant in the univariate model. All P values were two-tailed, and the alternative hypothesis was considered significant if P ≤ 0.05. Missing data was omitted from the analysis without imputations. All statistical analysis was done using SPSS software, version 26 (SPSS, Armonk, NY: IBM Corp).

## Results

**Fig. 1:**
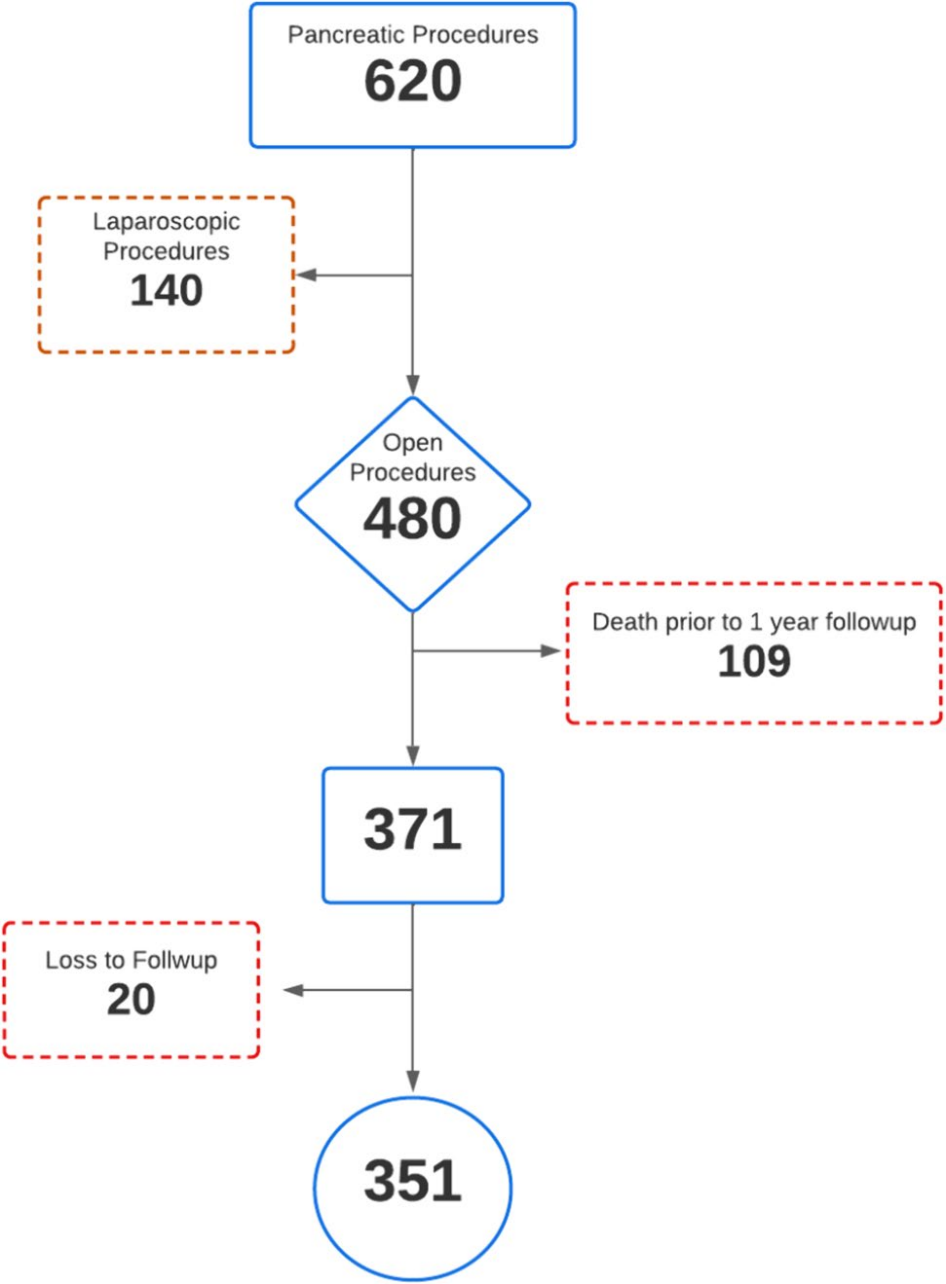
Selection Process Flowchart Depicting the Inclusion Criteria for the Study Cohort

Between January 2008 and December 2021, a total of 620 patients underwent pancreatic surgery, with 480 (77.4%) of these procedures performed using an open approach. Of these, 109 patients (22.7%) died within the 1st year after surgery, and 20 (4.1%) were lost to follow-up (Fig. 1).

Of the remaining 351 patients, 208 (59.2%) underwent PD, 128 (36.4%) underwent LP, 10 (2.8%) who had a total pancreatectomy, and 5 (1.4%) underwent other types of pancreatic surgeries.

Of the entire cohort, 61 patients (17.38%) developed IH at least 1 year after surgery (Table 1), and during a mean follow-up period of 794 days (IQR 1694–537). Of these 61, 26 (42.6%) patients had symptoms related to the hernia reported as worsening quality of life. In 55 (90.1%) patients IH was evident in physical examination. In 25 (40.9%) patients IH was confirmed by cross sectional imaging, and in 5 (8.2%) IH was identified only by imaging. Of the entire cohort, patients who developed IH were older (mean age 68.7 vs. 64.9 years, p = 0.015), had higher body mass index (BMI) (27.7 ± 4.26 kg/m2 vs. 26.03 ± 4.67 kg/m2, p = 0.011), and had higher rates of chronic obstructive pulmonary disease (COPD) and history of cerebrovascular accident (CVA), as well as lower rates of chronic renal failure (CRF) (Table 1). Patients who underwent preoperative stenting of the CBD via endoscopic retrograde cholangiopancreatography (ERCP) also had higher rates of IH (Table 1), yet the preoperative Bilirubin levels were comparable between the groups (Table 1). Of note, the time period between stent placement and surgery was comparable between those who developed IH and those who did not (62.6 ± 98.2 days vs. 103.7 ± 113.1 days, p = 0.17). Patients who received neoadjuvant chemotherapy had a lower rate of IH compared to those who did not (Table 1). Preoperative serum albumin levels as well as rates of diabetes and active smoking did not differ significantly between the groups. The underlying pathology was comparable between those who developed IH and those who did not (Table 1). Of patients with PDAC, the rate of those who received adjuvant chemotherapy after surgery was comparable between the groups (Table 1).

**Table 1:** Correlation between IH and demographic and comorbidity variables in all pancreatic procedures

	All patients n = 351	IH n = 61	No IH n = 290	*P*
Age, mean ± SD	65.4 ± 11.82	68.7 ± 10.53	64.9 ± 11.1	0.015
Female, n (%)	176 (50.14)	33 (54.1)	143 (49.3)	0.93
Mean BMI (kg/m^2^)	26.32 ± 4.65	27.7 ± 4.26	26.03 ± 4.67	0.011
Diabetes, n (%)	119 (33.9)	23 (37.7)	96 (33.1)	0.685
HTN, n (%)	159 (45.3)	37 (60.7)	122 (42.1)	0.99
IHD, n (%)	23 (6.55)	5 (8.2)	18 (6.2)	0.154
COPD, n (%)	10 (2.8)	3 (4.9)	7 (2.4)	< 0.001
CRF, n (%)	16 (4.56)	1 (1.6)	15 (5.2)	< 0.001
Preoperative Creatinine (mg/dL), mean ± SD	0.9 ± 0.26	0.9 ± 0.28	0.9 ± 0.25	0.94
Asthma, n (%)	16 (4.56)	2 (3.3)	14 (4.8)	0.095
CVA, n (%)	7 (1.99)	2 (3.3)	5 (1.7)	< 0.005
Active smoker, n (%)	93 (26.5)	16 (26.2)	77 (26.6)	0.944
Preoperative Albumin (g/dL), mean ± SD	3.7 ± 0.6	3.77 ± 0.6	3.7 ± 0.61	0.33
Neoadjuvant chemotherapy, n (%)	65 (18.5)	6 (9.8)	59 (20.3)	0.006
Preoperative CBD stenting, n (%)	72 (20.5)	19 (31.1)	53 (18.3)	0.047
Preoperative Bilirubin (mg/dL), mean ± SD	2.85 ± 5.03	2.9 ± 5.2	2.84 ± 5	0.99
PDAC, n (%)	219 (62.4)	39 (63.9)	180 (62.1)	0.99
NET, n (%)	31 (8.83)	6 (9.83)	25 (8.62)	0.47
IPMN, n (%)	27 (7.69)	5 (8.2)	22 (7.58)	0.64
Other pathologies*, n (%)	68 (19.37)	11 (18.03)	57 (19.65)	0.82
Adjuvant chemotherapy, n (%)	183 (52.1)	27 (44.2)	156 (53.8)	0.94

*BMI* Body mass index, *HTN* Hypertension, *IHD* Ischemic heart disease, *COPD* Chronic obstructive pulmonary disease, *CRF* Chronic renal failure, *CBD* Common bile duct, *CVA* Cerebrovascular Accident, *PDAC* Pancreatic Ductal Adenocarcinoma, *IPMN* Intraductal Papillary Mucinous Neoplasms, *MCN* Mucinous Cystic Neoplasm

^*^Other pathologies include—Autoimmune pancreatitis, chronic pancreatitis, metastasis from renal cell carcinoma, serous cystadenoma, Liposarcoma, other sarcoma, Solid Pseudo papillary neoplasm

Significant predictors for the development of IH in a binary logistic regression analysis included perioperative CBD stenting (OR 2.05; 95% CI 1.06–3.96; p = 0.03), increased age at diagnosis (1-year- increment; OR 2.05; 95% CI 1.06–3.96; p = 0.01), and BMI (OR 1.08; 95% CI 1.01–1.15; p = 0.01) (Table 2). Although COPD, CRF, CVA and neoadjuvant chemotherapy treatment had a significant correlation with IH, the association did not remain significant in the multivariate analysis.

**Table 2:** Demographics and pre-operational predictors for incisional hernia within the cohort

Variable	Univariate OR (95%CI)	Multivariate OR (95%CI)	*P*	Wald	S.E
Preoperative CBD stenting, n (%)	2.02 (1.09 to 3.75)	2.05 (1.06 to 3.96)	**0.03**	4.53	0.34
Age, 1-year-increment	1.035 (1.01 to 1.06)	1.04 (1.01 to 1.07)†	**0.01**	6.06	0.02
BMI	1.08 (1.02 to 1.14)	1.08 (1.01 to 1.15)	**0.01**	5.92	0.03
COPD, n(%)	2.09 (0.53 to 8.33)	1.93 (0.44 to 8.47)	0.38	0.77	0.75
CRF, n(%)	0.31 (0.4 to 2.36)	0.21 (0.02 to 1.68)	0.14	2.16	1.05
CVA, n(%)	1.93 (0.37 to 10.20)	1.60 (0.26 to 9.58)	0.60	0.26	0.91
Neoadjuvant chemotherapy, n(%)	0.43 (0.18 to 1.04)	0.44 (0.18 to 1.12)	0.08	2.94	0.47

*S.E* standard error* CBD* Common Bile Duct, *BMI* Body mass index, *COPD* Chronic obstructive pulmonary disease, *CRF* Chronic renal failure, *CVA* Cerebrovascular Accident,

¥ Obtained from a univariate binary logistic regression model

We further assessed whether postoperative complications were related to higher rates of IH. When evaluating all patients, those with IH had significantly increased rates of surgical site infections (SSI), severe postoperative complications (Clavien-Dindo > 3a), and reoperations compared to those who did not develop IH (Table 3). Of note, the rates of pancreatic fistula, including high grade fistula, was comparable between the groups (Table 3).

**Table 3:** Correlation between IH and surgical complications in all pancreatic procedures

	All patients n = 351	IH n = 61	No IH n = 290	*P*
SSI, n (%)	31 (8.83)	10 (16.4)	21 (7.2)	< 0.001
Pancreatic Fistula, n (%)	129 (35.75)	25 (41.0)	104 (35.9)	0.717
Pancreatic Fistula ≥ Grade 2, n (%)	71 (20.2)	15 (24.6)	56 (19.3)	0.32
Clavien-Dindo > 3a, n (%)	40 (11.4)	10 (16.4)	30 (10.3)	0.003
Readmission, n (%)	143 (40.7)	29 (47.5)	114 (39.3)	0.74
Re-operation, n (%)	39 (11.1)	11 (18.0)	28 (9.7)	0.004

*SSI* Surgical site infection

Using the binary logistic regression analysis model among postoperative constellations, SSI was the only significant variable associated with IH (OR 2.86; 95% CI 1.24–6.57; p = 0.01). Although a Clavien-Dindo score above 3a, and a need for re-operation had a significant correlation with IH the association did not remain significant in the multivariate analysis (Table 4).

**Table 4:** Post-operational predictors for incisional hernia within the cohort

	Univariate OR (95%CI)	Multivariate OR (95%CI)	P-value	Wald	S.E
SSI, n (%)	2.51 (1.12 to 5.65)	2.86 (1.24 to 6.57)	**0.01**	6.13	0.42
Clavien-Dindo > 3a, n (%)	1.80 (0.79 to 4.10)	1.17 (0.34 to 3.96)	0.80	0.06	0.62
Re-operation, n (%)	2.06 (0.96 to 4.40)	1.97 (0.57 to 6.76)	0.27	1.17	0.62

¥ Obtained from a univariate binary logistic regression model

*SSI* Surgical site infection, *S.E* standard error

Of the entire cohort, 72 patients (20.5%) underwent preoperative stenting of the CBD. Generally, those who underwent preoperative stenting had higher rates of SSI and IH (Table 5). The rate of those who had a documented SSI and later developed IH was also higher among those who underwent CBD stenting. Of note, no significant difference in rates of patients who developed IH without SSI was noted between patients who underwent CBD stenting and those who did not.

**Table 5:** Rates of SSI and IH in relation to preoperative stenting of the CBD in all patients

	Preoperative CBD stenting n = 72	Without stent n = 279	*P*
IH, n %	19 (26.4)	42 (15.1)	0.016
SSI, n %	19 (26.4)	12 (4.3)	< 0.001
SSI + IH, n %	7 (9.7)	3 (1.1)	< 0.001
IH without SSI, n %	12 (16.6)	39 (13.9)	0.384

*IH* Incisional hernia, *SSI* Surgical site infection

The rate of IH was markedly higher in those who underwent PD compared to those who underwent LP (20.19% in the PD versus 12.1% in the LP group). Importantly, SSI was documented only after PD. In our cohort, none of the patients who underwent LP had a documented SSI after surgery. In light of this data we assessed groups, those who underwent PD and those who underwent LP, separately.

When only PD was examined, patients who developed IH were older, and had increased rate of prior CVA (Table 6). Those who received neoadjuvant therapy had significantly lower rates of IH (Table 6). Moreover, PD patients that suffered from SSI, clinically significant pancreatic fistula (> grade 2), major postoperative complications (Clavien-Dindo > 3a), and reoperations had significantly higher rates of IH (Table 6).

**Table 6:** Correlation between IH and demographic and comorbidity variables in patients who underwent PD

	All PD patients n = 208	IH n = 42	No IH n = 166	*P*
Age, mean ± SD	67.13 ± 10.36	70.2 ± 10.26	66.3 ± 10.24	0.003
Female n %	102 (49)	22 (52.3)	80 (48.19)	0.9
Mean BMI (kg/m^2^)	26.4 ± 4.4	27 ± 4.18	26.2 ± 4.43	0.36
Diabetes, n (%)	69 (33.2)	16 (38.10)	53 (31.93)	0.64
HTN, n (%)	93 (44.7)	25 (59.52)	68 (40.96)	0.91
IHD, n (%)	15 (7.21)	4 (9.52)	11 (6.63)	0.13
COPD, n (%)	4 (1.92)	1 (2.38)	3 (1.81)	0.21
CRF, n (%)	9 (4.3)	0	9 (5.42)	–
Asthma, n (%)	11 (5.3)	0	11 (6.63)	–
CVA, n (%)	6 (2.9)	2 (4.76)	4 (2.41)	0.003
Active smoker (%)	57 (27.4)	8 (19.05)	49 (29.52)	0.29
Neoadjuvant chemotherapy, n (%)	35 (16.8)	3 (7.14)	32 (19.28)	0.002
Preoperative CBD stenting, n (%)	70 (33.6)	17 (40.48)	53 (31.93)	0.585
SSI, n (%)	28 (13.5)	9 (21.43)	19 (11.45)	0.022
Pancreatic Fistula, n (%)	117 (56.25)	71 (16.05)	46 (27.71)	0.37
Pancreatic Fistula > Grade 2, n (%)	29 (13.9)	9 (21.4)	20 (12.05)	0.003
Clavien Dindo > 3a, n (%)	23 (11.1)	7 (16.67)	16 (9.64)	0.035
Readmission, n (%)	84 (40.4)	20 (47.6)	64 (38.55)	0.74
Re-operation, n (%)	19 (9.1)	7 (16.67)	12 (7.23)	0.001
PDAC, n (%)	152 (73)	31 (73.8)	121 (72.9)	0.97

*BMI* Body mass index, *HTN* Hypertension, *IHD* Ischemic heart disease, *COPD* Chronic obstructive pulmonary disease, *CRF* Chronic renal failure, *CVA* Cerebrovascular accident, *CBD* Common bile duct, *SSI* Surgical site infection, *PDAC* Pancreatic Ductal Adenocarcinoma

Of note, when assessing only patients who underwent PD, no direct correlation was observed between preoperative CBD stent placement and the formation of IH. However, the incidence of SSI was significantly higher in the stent group (27.1% vs. 6.5%, p < 0.001), and patients with SSI in this subgroup had a higher incidence of IH when compared to the non-stent group (10.0% vs. 1.4%, p < 0.001) (Table 7).

**Table 7:** Rates of SSI and IH in relation to preoperative stenting of the CBD in PD patients

	Preoperative CBD stenting n = 70	Without stent n = 138	*P*
IH, n %	17 (24.3)	25 (18.1)	0.27
SSI, n %	19 (27.1)	9 (6.5)	< 0.001
SSI + IH, n %	7 (10)	2 (1.4)	< 0.001
IH without SSI, n %	10 (14.2)	23 (16.6)	0.83

*IH* Incisional hernia, *SSI* Surgical site infection

Within the group of patients that underwent LP, Increased BMI and rates of COPD and asthma were the only factor that were significantly different between those who developed IH and those who did not. Unlike patients who underwent PD, in those who underwent LP the rate of postoperative complications such as SSI, pancreatic fistula, major postoperative complications, and reoperation were not significantly different between those who developed IH and those who did not (Table 8).

**Table 8:** Correlation between IH and demographic and comorbidity variables in patients who underwent LP

Category	All DPS patients n = 128	IH n = 15	No IH n = 113	*P*
Age	63.1 ± 11.9	63.9 ± 10.96	63.0 ± 12.07	0.78
Female, n %	66 (52)	8 (53.33)	56 (51.85)	0.8
Mean BMI (kg/m^2^)	26.23 ± 5.08	30.57 ± 3.6	25.6 ± SD4.9	0.005
Diabetes, n %	43 (35)	5 (33.33)	38 (35.19)	0.85
HTN, n %	56 (45.5)	9 (60)	47 (43.52)	0.84
IHD, n %	7 (5.7)	1 (6.67)	6 (5.56)	0.49
COPD, n %	6 (4.9)	2 (13.3)	4 (3.7)	0.0002
CRF, n %	6 (4.9)	1 (6.7)	5 (4.6)	0.249
Asthma, n %	4 (3.25)	2 (13.3)	2 (1.85)	< 0.001
CVA, n %	1 (0.81)	0	1 (0.93)	–
Active smoker, n %	32 (26.02)	7 (46.67)	25 (23.15)	0.25
Neoadjuvant, n %	28 (22.8)	2 (13.3)	26 (24.07)	0.4
Preoperative CBD Stent, n %	1 (0.81)	1 (6.7)	0	–
SSI, n %	2 (1.63)	0	2 (1.85)	–
Pancreatic Fistula, n %	61 (49.6)	8 (53.3)	53 (49.1)	0.8
Pancreatic Fistula > Grade 2, n %	38 (30.9)	6 (40)	32 (29.6)	0.5
Clavien Dindo, n % > 3a	14 (11.4)	3 (20)	11 (10.2)	0.07
Readmission, n %	54 (43.9)	9 (60)	45 (41.7)	0.82
Re-operation, n %	18 (14.6)	4 (26.7)	14 (12.96)	0.08
PDAC, n %	59 (47.97)	5 (33.3)	54 (50)	0.96

*BMI* Body mass index, *HTN* Hypertension, *IHD* Ischemic heart disease, *COPD* Chronic obstructive pulmonary disease, *CRF* Chronic renal failure, *CVA* Cerebrovascular accident, *CBD* Common bile duct, *SSI* Surgical site infection, *PDAC* Pancreatic Ductal Adenocarcinoma

Compared to the PD group, the rates of preoperative stenting and SSI were much lower in the LP group (One patient underwent preoperative stenting of the CBD, and two patients developed SSI) and hence they did not significantly correlate with development of IH.

Patients with asymptomatic IH were followed conservatively. Those with symptomatic IH but with evidence of recurrence of malignancy were also treated conservatively. Those with symptomatic IH affecting the quality of life with no evidence of malignancy underwent surgical repair of their IH. None of the patients required emergent operation for IH incarceration.

## Discussion

In this report, we conducted a retrospective analysis of factors related to the development of IH in patients who underwent pancreatic surgery over a 14-year period. We assessed the occurrence of IH as identified either by physical examination, or by imaging, starting from 1 year after surgery. Patients with asymptomatic defects were also identified since all patients who underwent pancreatic resection in this cohort had a routine cross-sectional imaging follow-up. Overall, the rate of 17.38% IH 1 year after surgery stands in good agreement with other reports of IH in elective midline laparotomy cases without the use of a prophylactic mesh [8, 9]. We show that when including all patients in our cohort, older age, higher BMI and other comorbidities such as COPD were associated with higher rates of IH. This is not unique to pancreas surgery and was previously descried in numerous reports [10, 11]. Specific factors related to pancreatic surgery such as neoadjuvant therapy for PDAC, preoperative CBD stenting, and specific pancreatectomy related complications were also evaluated. Increased age, Higher BMI, and preoperative CBD stenting were significant predictors in a multivariable analysis. We show that patients who received neoadjuvant treatment had lower rates of IH. This did not remain significant in a multivariable model. Previous non-pancreatic cancer studies have shown higher rates of IH in patients who received adjuvant and neoadjuvant chemotherapy [12]. In these studies, many of the patients received anti-angiogenic factors such as Bevacizumab, and the effect was noted especially in these who received many courses. However, the trend towards a protective effect of neoadjuvant therapy documented in our cohort is not completely clear and requires further investigation.

SSI, pancreatic fistula, major postoperative complications, and reoperations were associated with increased rates of IH when assessing all patients. Of these, SSI was the only predictor that remained statistically significant in a multivariable model. Since the rate of these factors differ significantly between PD and LP we further evaluated each of these surgeries separately. Indeed the effect of these factors on IH was mainly significant within the PD group. The enteric anastomosis (which requires a controlled opening of a contaminated space), which is inherent to PD, categorizes it as a clean-contaminated operation, in contrast to LP, which is generally considered a clean procedure. This distinction is reflected in our study's SSI rates, where the PD group exhibited an overall SSI rate of 13.46%, as opposed to the negligible SSI occurrences in the LP group. Naturally, most of the patients who underwent preoperative CBD stenting had a lesion in the head of the pancreas that necessitated a PD (Only one patient who underwent LP had preoperative CBD stenting). Hence, in the PD group, preoperative CBD stenting was associated with increased rates of SSI, which in turn was associated with higher rates of IH, whereas in the LP, almost no CBD stenting and SSI indeed resulted in lower rates of IH.

In our cohort, the rate of pancreatic fistula was higher in those who underwent LP compared to PD patients, as shown in various reports. However, within the PD, but not in the LP group, the clinically significant pancreatic fistula was associated with higher rates of IH. This may be related to the increased likelihood of pancreatic fistulas after PD to be infected compared with those after DPS.

Identifying these risk factors may pave the way for targeted preventive strategies in selective cases, which might enhance surgical outcomes. One such preventive strategy, designed to thwart incisional hernias, is the worldwide adoption of a prophylactic mesh implant during permanent colostomy surgeries [13].

## Limitations

The retrospective design of this study inherently limits the scope of our conclusions, particularly concerning the clinical significance of the postoperative ventral hernias identified. The impact of these hernias on patients' quality of life was not directly assessed, and it is possible that some hernias, although radiologically apparent, may have been clinically asymptomatic and thus of uncertain significance. The study's setting in a single, high-volume center may affect the generalizability of the findings to other healthcare environments. Additionally, due to the observational nature of the research, the identified associations cannot be interpreted as causal. Prospective studies are needed to further explore the clinical relevance of incisional hernias post-pancreatic surgery and to rigorously test the efficacy of any proposed prophylactic interventions. Importantly, all pancreatectomies in this study were performed via midline incision. Pancreatic resection can also be performed via transvers incision, also known as "Chevron incision". Although transverse abdominal incisions were shown to be associated with lower rates of incisional hernia, Chevron incision necessitate complete transection of the rectus abdominis muscles and innervation. This can lead to severe abdominal wall muscle atrophy which in turn associated with significant patient morbidity and impact on quality of life [14]. Importantly, this pathology cannot be fixed with surgery. Hence, it is our long standing preference to perform all our open pancreatectomies via midline laparotomy. However, this of course limits our findings to this incision only.

## Conclusions

We described the incidence and related factors to the development of IH following PD and LP. We show that preoperative stenting of the CBD increases the likelihood of SSI which in turn is associated with IH. We also show that clinically significant pancreatic fistula after PD but not after LP is associated with higher rates of IH. If further research supports our findings, the increased risk associated with stent placement and the high likelihood of developing post-pancrectomy pancreatic fistula may justify the consideration of preventive measures, such as the prophylactic mesh placement.

## Data Availability

Not applicable.
